# Exosomes: Roles and Therapeutic Potential in Pain

**DOI:** 10.3390/biomedicines14020414

**Published:** 2026-02-11

**Authors:** Yuting Wen, Rui Zhang, Jitong Wang, Zhouyun Ma, Changsheng Dong, Ruixiang Li, Jiange Zhang

**Affiliations:** 1 Innovation Research Institute of Traditional Chinese Medicine (IRI), Shanghai University of Traditional Chinese Medicine, Shanghai 201203, China; wenyuting@shutcm.edu.cn (Y.W.); rui_zhang@shutcm.edu.cn (R.Z.); wangjitong@shutcm.edu.cn (J.W.); mjf2mjf@163.com (Z.M.); 2 Cancer Institute of Traditional Chinese Medicine, Longhua Hospital, Shanghai University of Traditional Chinese Medicine, Shanghai 200032, China

**Keywords:** exosomes, inflammatory pain, neuropathic pain, cancer pain, drug delivery

## Abstract

Pain is a signal that the human body is being damaged or attacked by disease. It significantly impacts quality of life and imposes a substantial economic burden. Current analgesic drugs fail to meet clinical application standards due to limited choice, inadequate efficacy, and side effects. Consequently, the development of new treatment strategies for pain relief is essential. Pain signals are conveyed by nociceptors via the central nervous system to the brain, with cell-to-cell communication serving as a crucial step in the sensory nociceptive process. Exosomes are extracellular vesicles involved in intercellular communication, capable of transporting and delivering biological macromolecules. Growing evidence suggests that exosomes contribute significantly to the pathological processes associated with pain-related diseases. Summarizing the characteristics of exosomes and their molecular cargo under various pain conditions, along with identifying specific exosomal signatures, is essential for the early diagnosis and treatment of such diseases. This review systematically elucidates the molecular and cellular mechanisms of exosomes in pain relief and evaluates their potential therapeutic value in pain management. We aim to deepen the understanding of exosome–pain interactions, thereby laying the foundation for developing novel and promising therapeutic strategies. Furthermore, we scrutinize the current status of clinical research on exosome-mediated analgesia and dissect the prevailing technical challenges and future research directions. Our objective is to provide clear scientific guidance and a theoretical basis to facilitate the clinical translation of exosome therapies.

## 1. Introduction

Pain is characterized as an unpleasant sensory and emotional experience linked to actual or potential tissue damage. It has emerged as the third leading health issue following cardiovascular disease, cerebrovascular diseases, and tumors [1]. Pain is projected to impact about 20% of the worldwide population, resulting in significant health and economic consequences [2]. Pain acts as a critical mediator between primary diseases and secondary pathological outcomes. This can lead to a complex and harmful interaction among biological, social, and psychological factors, imposing disability and unfavorable outcomes for patients. Analgesic drugs serve as the primary approach for managing both acute and chronic pain. Although effective in the short term, there are considerable concerns about drug dependence, addiction, and other adverse effects [3,4]. In addition to the creation of innovative analgesic drugs to reduce pain, emerging therapeutic approaches may offer significant benefits in pain management.

Pain signals are conveyed by nociceptors via the central nervous system to the brain, with cell-to-cell communication being an essential step in the sensory nociceptive process [5]. Recent years have seen a growing interest in extracellular circulation vesicles and their involvement in various pain states, particularly exosomes, which have garnered the most focus. Exosomes are present in various bodily fluids, secreted by diverse cell types, and they participate in information exchange between cells by carrying signaling substances [6]. In addition, exosomes can be administered locally or systemically, exhibit low tumorigenicity and immunogenicity, and serve as potential vectors and therapeutic agents for pain regulation.

Exosomes are nanoscale vesicles derived from endosomes that fuse with the plasma membrane, mediating the transfer of various cargoes, such as proteins, lipids, nucleic acids, and other active substances, to the extracellular environment or target cells [7]. Exosomes are generated by nearly all cell types in mammals and can be identified in various body fluids, including blood, urine, joint fluid, saliva, and breast milk. This highlights their function as mediators of intercellular communication and their involvement in both pathological and physiological disease processes, demonstrating a significant capacity for transferring [8,9]. Studies have demonstrated the involvement of exosomes in the pain process across various chronic pain disorders, particularly osteoarthritis (OA) [10], rheumatoid arthritis (RA) [11], inflammatory bowel disease (IBD) [12], and neurodegenerative and complex regional pain syndrome (CRPS) [13]. Evidence suggests that exosomes can alleviate pain symptoms with reduced adverse reactions and exhibit immunoprotective and anti-inflammatory properties [14]. Therefore, exosomes are regarded as a potential therapeutic option for pain. In addition, exosomes serve as a carrier tool that can benefit patients with a specific treatment, enabling precise diagnosis and treatment [15]. This state-of-the-art review provides a comprehensive synthesis of the molecular and cellular mechanisms of exosomes, emphasizing their therapeutic potential in pain management. Beyond summarizing foundational signaling pathways, this work critically evaluates current clinical advancements and the formidable bottlenecks hindering translational progress. By elucidating the sophisticated analgesic mechanisms of exosomes, we aim to provide a robust theoretical framework and evidence-based support for their integration into clinical practice.

## 2. Literature Search and Selection

A systematic literature search was conducted across the PubMed, Web of Science, X-mol, and Google Scholar databases, covering all relevant literature published up to October 2025. The search strategy employed “exosomes” or “extracellular vesicles” as core terms, logically combined with keywords including “pain”, “neuropathic pain”, “inflammatory pain”, and “cancer pain”. Inclusion criteria focused on studies utilizing exosomes or extracellular vesicles for pain intervention, while non-English publications and studies lacking sufficient experimental evidence were excluded. To ensure scientific rigor and timeliness, priority was given to original research articles providing clear exosome characterization in accordance with the International Society for Extracellular Vesicles guidelines. Furthermore, high-impact findings from the past five years (2020–2025) were emphasized to provide a comprehensive, in-depth, and high-quality academic landscape.

## 3. Exosomes

**Figure 1:**
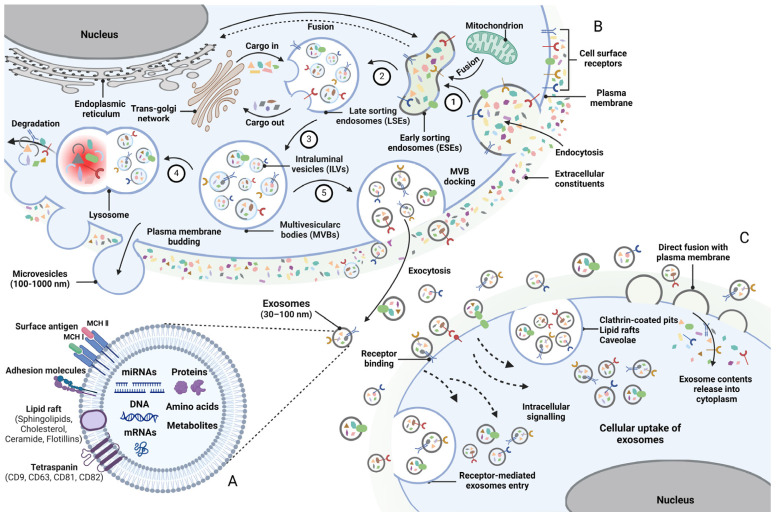
Biogenesis of exosomes. (**A**) Exosomes carry a diverse molecular cargo, including proteins and nucleic acids. (**B**) The biogenesis process involves the formation of the early sorting endosomes (ESEs) via endocytosis and plasma membrane invagination (Step 1); maturation into the late sorting endosomes (LSEs) (Step 2); the generation of MVBs containing intraluminal vesicles (ILVs) through secondary membrane invagination and cargo sorting (Step 3); mature MVBs are either targeted to lysosomes for enzymatic degradation (Step 4) or trafficked along the cytoskele-ton to the plasma membrane to secrete ILVs into the extracellular microenvironment (Step 5). (**C**) Exosomes carry cargoes to target cells through various mechanisms. (Created in Biorender. Wen, Y. (2026) https://BioRender.com/u59y127).

Extracellular vesicles (EVs) are tiny vesicles released into the extracellular environment via cell membrane fusion, following the formation of multivesicular bodies through endocytosis under both physiological and pathological conditions [16]. The International Society for Extracellular Vesicles classifies them into three types based on their biogenesis and size. These are microvesicles (MVs), exosomes, and apoptotic bodies, respectively. MVs and apoptotic bodies typically range in size from 100 to 1000 nm and 1 to 4 µm, respectively, and are released via plasma membrane budding [17]. In contrast, exosomes, measuring 30–150 nm in diameter, bud inward from late endosomes to form a multivesicular body, which subsequently fuses with the plasma membrane for release [13]. Exosomes and MVs share similar size (100–150 nm) and density (1.08–1.19 g/mL) characteristics, making differentiation challenging. Consequently, exosomes are typically identified by the presence of endosome-associated proteins, which include the tetraspanins CD9, CD63, and CD81 (Figure 1A) [18].

Exosomes are nanoscale extracellular vesicles that originate from endosomes and are released by almost all types of cells, including blood cells, immune cells, tumor cells, and stem cells. Once released, exosomes are easily distributed into the body’s internal environment, where they transfer biological signaling molecules, including proteins, lipids, and nucleic acids, from donor cells to recipient cells [19]. The chronic development of pain is the result of the interaction between neuronal cells, glial cells, and immune cells, suggesting that intercellular communication and signal transmission play an important role in the regulation of the nociceptive pain signal transduction pathway [20]. Exosomes serve as crucial agents in intercellular communication and play significant roles in diverse physiological and pathological processes. Exosomes are closely linked to various chronic pain conditions, exhibiting fewer adverse reactions while providing immune protection and anti-inflammatory effects [10,11,13].

## 4. Biogenesis of Exosomes

Exosomes, similar to liposomes, feature a lipid membrane and an internal aqueous environment; however, research has indicated that exosomes exhibit greater complexity, encompassing a higher concentration of proteins and lipids. Exosomes are generated within the endosomal compartment of most eukaryotic cells and are subsequently released into the extracellular environment following fusion with the plasma membrane [21]. Exosomes were initially considered to serve a role in lysosomal degradation, the recovery of cellular components, and the excretion of cellular waste [22]. However, recent studies have revealed that exosomes are of critical importance in various physiological and pathological processes within the body and exhibit diverse functions. Most cells are capable of releasing exosomes through endocytosis and biogenic synthesis pathways [23]. The endocytosis pathway commences upon the reception of external or internal signals from the surrounding environment. Afterwards, the cell membrane begins to invaginate, leading to the formation of the ESEs. The early endosome transitions to the LSEs under the regulation of multiple cellular signaling pathways. Multivesicular bodies (MVBs) created by LSEs merge with the cell membrane to release exosomes or are subjected to degradation (Figure 1B) [24,25,26].

The endosomal sorting complex required for transport (ESCRT) and the gene-2-interacting protein X, associated with apoptosis and tumor susceptibility gene 101, primarily facilitate the formation and release of exosomes [27,28,29,30]. The ESCRT complex primarily consists of ESCRT-0, ESCRT-I, ESCRT-II, and ESCRT-III [27]. ESCRT-0, -I, and -II are all composed of ubiquitin-binding subunits that have the ability to seize ubiquitin-tagged cargoes. On the other hand, ESCRT-III is involved in the process of vesicle budding and scission [31,32]. Nevertheless, under some circumstances, the production of exosomes might take place independently of the ESCRT complex of cells. Exosome production can be facilitated by the lipids. In a ceramide-activated model, for instance, the transfer of exosome-associated domains into the endosome lumen does not depend on the operation of the ESCRT [32]. Exosomes function as cell secretory vesicles that transport nucleic acids, proteins, and lipids in a paracrine or endocrine manner, thus modulating the biological functions of recipient cells [33]. Exosomes, upon release, convey information to recipient cells through various mechanisms, including fusion with plasma membranes, clathrin-coated pits, receptor-mediated entry, and lipid rafts (Figure 1C).

## 5. Exosomes and Pain

### 5.1. Exosomes and Inflammatory Pain

Inflammation represents an immune response by the body to infection or injury, aimed at restoring homeostasis within tissues. However, an uncontrolled inflammatory response can lead to disease and multiple pain reactions throughout the body [34]. Exosomes possess significant therapeutic potential in various inflammatory diseases, including OA, RA, IBD, and chronic pain, owing to their capacity to transport biological macromolecules like miRNAs and proteins [35,36,37]. Here, we summarize the roles of exosomes in different inflammatory pain conditions (Table 1). Arthritis is caused by inflammation, infection, degeneration, trauma and other inflammatory injuries, and occurs in the joints and surrounding tissues. Patients often suffer from long-term chronic pain due to joint injury, which can result in permanent disability in severe cases [38,39].

**Figure 2:**
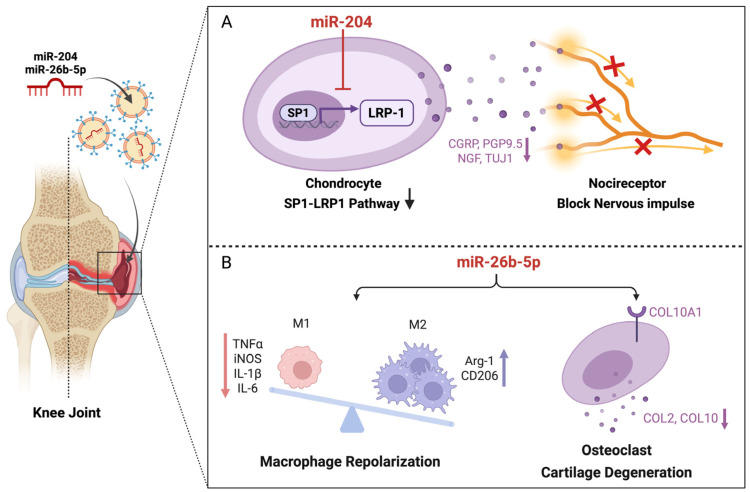
Exosomes relieve OA pain by inhibiting neuro-cartilage interactions within the joint. (**A**) miR-204 attenuates nociceptor invasion and suppresses neuronal markers via the SP1-LRP1 pathway. (**B**) miR-26-5p exerts dual analgesic and chondroprotective effects by promoting M2 macrophage polarization to reduce inflammation and inhibit COL10A1-mediated chondrocyte hypertrophy. (Created in Biorender. Wen, Y. (2026) https://BioRender.com/26rt2p5).

OA represents the most prevalent type of arthritis, marked by the degeneration of cartilage. Exosomes play a significant role as mediators of intercellular communication, contributing substantially to the onset and progression of OA, and exhibit considerable potential for therapeutic intervention in this condition [40]. Lu et al. examined the interactions between chondrocytes and neural cells, assessing the effect and mechanism of exosomes miR-204 in the treatment of OA pain. Their findings indicated that miR-204 may alleviate OA pain by downregulating low-density lipoprotein receptor-related protein 1 (LRP1) expression through the inhibition of transcription factor specificity protein 1 (SP1), thereby disrupting neuro-cartilage interactions within the joint. They loaded exosome simulators with miR-204 and injected them into the joint cavity of OA mice, and found that miR-204 significantly alleviated mechanical allodynia and improved the motion track-related indices in a destabilization of the medial meniscus (DMM) mouse model. In addition, miR-204 may suppress the expression of neuron makers to alleviate osteoarthritic pain through the inhibition of nociceptor sprouting (Figure 2A) [41]. Another study showed that intra-articular M2 macrophage-derived exosomes miR-26b-5p injection could improve gait behavioral index and cartilage degeneration for OA mice, and the underlying mechanism likely involved miR-26b-5p-mediated macrophage repolarization and the inhibition of chondrocyte hypertrophy by targeting toll-like receptor 3 (TLR3)/collagen type X alpha 1 (COL10A1) (Figure 2B) [42]. Furthermore, He et al. demonstrated that chondrocytes can internalize exosomes derived from bone marrow mesenchymal stem cells (BMSCs), leading to reduced interleukin-1β (IL-1β) levels, enhanced chondrocyte proliferation and migration, and significant promotion of extracellular matrix synthesis. These exosomes also alleviated knee pain and facilitated cartilage repair in osteoarthritic rats [43]. Moreover, subsequent research revealed that dental pulp stem cell-derived exosomes inhibited transient receptor potential vanilloid 4, thereby improving abnormal subchondral bone remodeling and attenuating cartilage degradation and synovial inflammation in vivo [44]. These studies suggested that exosomes are a promising treatment for OA pain.

For other inflammatory pain, Marguerite et al. analyzed lipopolysaccharide-induced macrophage-derived exosomes and serum-derived exosomes from patients with CRPS. Their results showed significant changes in inflammation-associated miR-126-5p, miR-21-3p, and miR-212, suggesting that exosomes play a role in dysregulated inflammation and chronic pain states [14]. In addition, human umbilical cord mesenchymal stem cells (HUC-MSCs)-derived exosomes containing miR-146a-5p improve the expression of autophagy-related proteins and inhibit the NOD-like receptor thermal protein domain-associated protein 3 (NLRP3) inflammasome in the spinal dorsal horn through the regulation of TNF receptor-associated factor 6 (TRAF6), thus mitigating neuroinflammation and reducing the inflammatory pain caused by complete Freund’s adjuvant (CFA) [45]. Ni et al. demonstrated that miR-134 played a role in CFA-induced inflammatory pain by balancing the expression of opioid receptor mu 1 (OPRM1) in dorsal root ganglions (DRGs) [46]. In addition, exosomes also have therapeutic effects against cystitis-induced bladder pain. The study demonstrated that miR-9-enriched mesenchymal stem cell (MSC)-derived exosomes mitigate neuroinflammation and cystitis-induced bladder pain by inhibiting the toll-like receptor 4 (TLR4)/nuclear factor kappa-B (NF-κB)/NLRP3 signaling pathway in interstitial cystitis mice [47]. Exosomes have also been implicated in IBDs, a collection of chronic diseases resulting from disruptions in gut homeostasis. One pathogenesis of IBDs involves the immune response regulated by macrophages. Exosomes derived from macrophages play a role in the pathogenesis of IBDs through immunosuppression [48]. The attenuation of experimental colitis by pioglitazone is dependent on the cleavage of annexin A1 released by macrophages. Interestingly, serum analysis of patients with IBDs revealed an elevated amount of exosomes containing membrane connexin-1, a protein crucial for mucosal repair and known to be overexpressed during IBD-related inflammation [49].

**Table 1:** Summary of exosomes and inflammatory pain.

Donor Cells	Cargo	Animals	Disease Model	Recipient Cells	Targets/Signaling Pathway	Ref.
Huc-MSCs	miR-146a-5p	Mouse	CFA-induced	BV2	TRAF6	[45]
RAW264.7	miR-126-5p miR-21-3p miR-212	Mouse	CFA-induced	-	-	[14]
-	miR-134	Rat	CFA-induced	DRG	OPRM1	[46]
BMSCs	-	Rat	OA	-	-	[43]
MSCs	miR-9	Mouse	Cystitis	Neuroglial cell	TLR4	[47]
MSCs	miR-204	Mouse	DMM	chondrocyte	SP1-LRP1	[41]
Macrophage	miR-26b-5p	Mouse	OA	-	TLR3/COL10A1	[50]

### 5.2. Exosomes and Neuropathic Pain

Neuropathic pain (NP) is a chronic secondary pain that is the result of a lesion or disease of the peripheral or central nervous system [51]. The development and maintenance of neuropathic pain are associated with interactions between neurons and glial cells, as well as between neurons and immune cells. Exosomes containing various cargoes function as key regulators that synchronize immune and neuronal activities, indicating their role in the pathological mechanisms of neuropathic pain (Table 2) [52,53]. Both the release and reuptake of exosomes by neurons depend on synaptic activity, and reports of their interneuronal communication suggested the importance of exosomes in neuropathic pain states [54]. Exosome cargos comprise various miRNAs, and recent findings demonstrated notable dysregulation of miRNAs in the DRGs and spinal cord following nerve injury [55,56,57]. These miRNAs can influence nociception; for example, intrathecal administration of miR-124, miR-103, and miR-23b reduces inflammatory and neuropathic pain by modifying the intracellular activities of neurones, astrocytes, and microglia [45,58,59].

**Figure 3:**
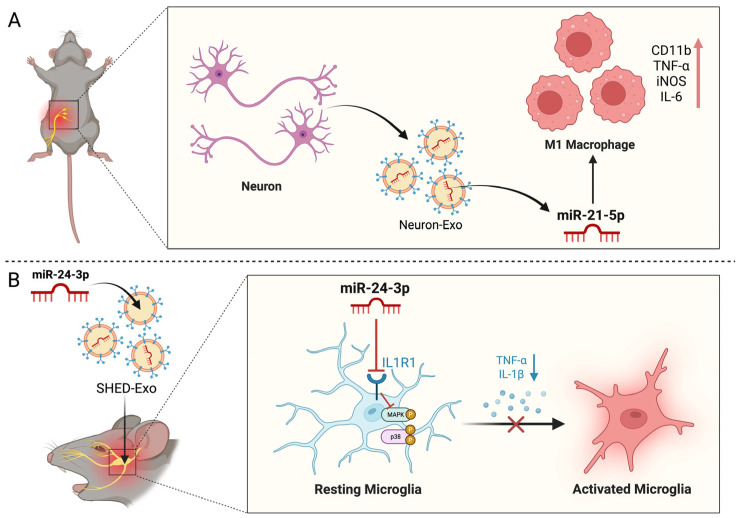
Exosomes relieve neuropathic pain in NP and CCI-ION models. (**A**) In SNI models, DRG neuron-derived exosomal miR-21-5p drives macrophage polarization toward a pro-inflammatory state. (**B**) SHED-Exos carrying miR-24-3p alleviate CCI-ION-induced pain by targeting the IL1R1/p-p38 MAPK axis. This action inhibits microglial activation and reduces the release of inflammatory factors in the STN mice. (Created in Biorender. Wen, Y. (2026) https://BioRender.com/6kppe12).

DRGs serve as the primary afferent nodes in the pain pathway. By integrating and transmitting signals from sensitized peripheral tissues to the central nervous system, they contribute significantly to the pathogenesis of NP [60]. The neuron–neuron cross-excitation that is mediated by miR-let7b is responsible for the pronociceptive effect. The miR-let7b, after being released by DRG neurones as a result of their activity, activates TRPA1 channels, which, in turn, provide sensory neurones with positive feedback [61]. Similarly, miR-134, expressed in DRGs, exhibits pronociceptive properties in chronic pain models, while the miR-183 cluster regulates genes associated with neuropathic pain in DRGs [46,62]. Simeoli et al. showed that DRG neuron cell bodies release exosomes, including containing miR-21-5p, upon activity after nerve injury. Macrophage phagocytosis of exosomes containing miR-21-5p results in phenotypic transformation, thereby sensitizing nociceptive neurons (Figure 3A). However, intrathecal miR-21-5p antagonist injection can block pain hypersensitivity in spared nerve injury (SNI) mice [63]. Interestingly, Zhang et al. also investigated the mechanism by which miR-21-5p reduces neuropathic pain, revealing that miR-21 expression is elevated in DRG neurones following spinal nerve ligation (SNL). This expression was found to colocalize with toll-like receptor 8 (TLR8) in small- and medium-sized neurones, but not in large neurones. Finally, their results revealed that miR-21 reduces neuronal excitability by regulating TLR8, which, in turn, mitigates mechanical allodynia in SNL mice [64]. In addition, exosomes carrying miR-16-5p released from DRGs facilitated microglial activation and enhanced the production of pro-inflammatory cytokines IL-1β and Interleukin-6 (IL-6) through the HECT domain E3 ubiquitin protein ligase 1 (HECTD1)-mediated ubiquitination of heat shock protein 90α, thereby exacerbating the SNL-induced neuropathic pain in mice [65].

NP states involve the long-term sensitization of spinal cord neurones that transmit nociceptive signals to the brain, resulting in significant activation of glial cells, such as astrocytes and microglia, within the spinal cord. This activation leads to the upregulation of proinflammatory cytokines and chemokines, which contribute to neuropathic pain [66,67,68,69]. Lu’s research indicated that miR-146a-5p may reduce neuropathic pain by partially decreasing TRAF6 and its downstream signaling pathway involving c-Jun N-terminal kinase and C-C motif chemokine ligand 2 in spinal glial cells [45]. Another study indicated that exosomes derived from Huc-MSCs produced analgesic effects on neuropathic pain by inhibiting the activation of the toll-like receptor 2 (TLR2)/myeloid differentiation primary response protein 88 (MyD88)/NF-κB signaling pathway in the spinal microglia. The mechanism underlying these antinociceptive effects involved exosome-mediated interference with the radical S-adenosyl methionine domain-containing 2 (Rsad2) expression, which subsequently inhibited microglial activation [70]. Similarly, BMSC-derived exosomal glial cell line-derived neurotrophic factor (GDNF) inhibits the same TLR2/MyD88/NF-κB pathway, thereby alleviating pain in a CCI rat model [71]. Recent research found that the microglia-derived exosome miR-124-3p in the hippocampus regulates microglia polarization to reduce postoperative mechanical hyperalgesia in aged mice [50]. Furthermore, sphingosine-1-phosphate (S1P) functions as a pivotal molecular switch regulating exosome biogenesis and release, while also serving as a potent bioactive lipid mediator that directly activates glial cells and neurons [72]. Following nerve injury, intracellular S1P levels are elevated in spinal glial cells. This hyperactivation of S1P signaling drives the extensive release of exosomes loaded with algogenic cargos from glia, thereby exacerbating neuroinflammation and central sensitization, ultimately leading to persistent neuropathic pain [73,74].

Moreover, there is some evidence to suggest that exosomes are involved in neuropathic pain. For instance, autologous conditioned serum (ACS), which is rich in anti-inflammatory cytokines and growth factors, has been used to successfully treat trigeminal neuralgia [75]. Buchheit et al. reported that a single intrathecal injection of human conditioned serum (hCS) resulted in the prolonged inhibition of mechanical allodynia associated with paclitaxel chemotherapy-induced neuropathic pain (CIPN) in mice, without inducing motor impairment, potentially through a reduction in nerve conduction damage. Importantly, the removal of exosomes through high-speed centrifugation significantly reduced the pain relief produced by CS, indicating a crucial role of exosomes in the therapeutic effects of CS [76]. In addition, another investigation indicated that exosomes derived from stem cells of human exfoliated deciduous teeth (SHED) significantly reduce microglial activation and neuronal hyperactivity in the spinal trigeminal nucleus (STN) of mice subjected to chronic constriction injury of the infraorbital nerve (CCI-ION), potentially via the involvement of the miR-24-3p/Interleukin 1 receptor type 1 (IL1R1)/p38 mitogen-activated protein kinase (p38 MAPK) axis (Figure 3B) [77]. Beyond the aforementioned molecules, circular RNAs constitute another critical category of exosomal cargo. For instance, CircHIPK3 is highly expressed in the DRGs of diabetic patients suffering from neuropathic pain, and its upregulation is positively correlated with pain intensity. Intrathecal injection of CircHIPK3 shRNA has been shown to significantly alleviate neuropathic pain in diabetic rats. Further mechanistic studies have demonstrated that this effect is mediated by the interaction between circHIPK3 and miR-124, which leads to the negative regulation of miR-124 expression [78]. Zhang et al. revealed that pain hypersensitivity induced by nerve injury is closely associated with the upregulation of circAnks1a. Mechanistically, in the cytoplasm, circAnks1a functions as a molecular sponge for miR-324-3p and facilitates the nuclear translocation of YBX1 by promoting its interaction with transportin-1. Upon entering the nucleus, circAnks1a enhances the recruitment of *YBX1* to the vascular endothelial growth factor B (VEGFB) promoter, thereby upregulating VEGFB expression. This resultant elevation in VEGFB levels heightens neuronal excitability in the dorsal horn, suggesting that the circAnks1a/VEGFB axis represents a promising therapeutic target [79].

**Table 2:** Summary of exosomes and neuropathic pain.

Donor	Cargo	Animal	Disease Model	Recipient	Targets/Signaling Pathway	Ref.
-	miR-let-7	Mouse	Formalin	DRG	TLR7/TRPA1	[61]
-	miR-183	Mouse	SNI	-	CACNA2D1/2	[62]
DRG	miR-21-5p	Mouse	SNI	Macrophage	-	[63]
DRG	miR-16-5P	Mouse	SNL	BV2	HECTD1	[65]
-	miR-146a-5P	Mouse	SNL	Astrocyte	TRAF6	[45]
Huc-MSCs	-	Rat	CCI	BV2	Rsad2	[70]
BMSCs	GDNF	Rat	CCI	-	TLR2/MyD88/NF-κB	[71]
BV2	miR-124-3p	Mouse	Postoperative pain model	BV2	-	[50]
Astrocyte	S1P	Mouse	CCI/SNI	Astrocyte	-	[74]
SHED	miR-24-3p	Mouse	CCI-ION	BV2	IL1R1/p38 MAPK	[77]
-	CircHIPK3	Rat	Diabetic neuropathic pain model	-	-	[78]
-	circAnks1a	Rat	SNL	-	VEGFB	[79]
UCMSCs	-	Rat	SNL	Neuroglia cells	-	[37]
-	miR-124	Mouse	SNI	BV2	GRK2	[59]
	miR-103	Rat	SNL	-	CaV1.2	[58]

### 5.3. Exosomes and Cancer Pain

Severe pain is present in over 60% of individuals diagnosed with primary or metastatic bone cancer [80]. The mechanisms that contribute to bone cancer pain involve distinct tumorigenic elements [81,82]. Osteolytic tumor growth has been shown in experimental models of bone cancer pain to sensitize C-fiber nociceptors and dorsal horn neurons [83,84,85,86]. Moreover, Khasabova et al. demonstrated via in vitro models of cancer cell–neuron interactions that substances produced by cancer cells directly sensitize DRG neurons [87]. Tumor-derived exosomes contain parental tumor cell material and affect intracellular signaling pathways by transporting their contents to recipient cells, thus contributing significantly to tumor development [88]. Numerous studies indicate that exosomes, functioning as a medium for cellular communication, can promote bone metastasis of tumors [89,90,91,92,93,94]. Several studies have also shown that exosomes are associated with cancer pain (Table 3). Lysophosphatidic acid (LPA) enhances the proliferation of fibrosarcoma cells in vitro and serves as a pain-signaling molecule that activates LPA receptors on nociceptive neurons and satellite cells in DRGs [95,96,97,98]. Studies have shown that the binding of exosomes secreted by cancer cells NCTC 2472 and autotaxin promoted the production of LPA and then promoted calcium inflow to sensitize neuronal nociceptive receptors [99]. In addition, Bhattacharya et al. reported elevated expression of pain and metastasis-related genes, specifically matrix metalloprotease 1 and Thrombospondin-1, in hypoxic exosomes derived from human oral tongue squamous OSC-20 cells. These exosomes were found to induce mechanical allodynia and thermal hyperalgesia in a mouse model [100]. Studies also provide evidence that DRG neurons can take up let-7d-5p from non-small-cell lung cancer cell-derived exosomes, decrease the protein level of the target gene OPRM1, and enhance cancer-induced bone pain (CIBP) [101].

Gandla et al. performed a genome-wide screen for miRNAs differentially expressed in DRG neurons within a mouse model of bone metastatic pain. They identified miR-34c-5p as a critical pronociceptive miRNA and demonstrated that it modulates cancer pain via the targeting of the voltage-gated calcium channel 2.3 (Cav2.3) [102]. In addition, the results indicated that miR-135-5p could mitigate CIBP in mice by inhibiting astrocyte-mediated neuroinflammation and blocking the janus kinase-2 (JAK2)/signal transducer and activator of the transcription 3 (STAT3) signaling pathway. This suggests that the upregulation of miR-135-5p could serve as a therapeutic target in the treatment of CIBP [103]. Another independent study demonstrated that BMSCs-derived miR-5-9p suppresses neuroinflammation in the spinal cord of CIBP mice and alleviates pain-related behaviors. This effect is achieved by downregulating the expression of RE1-silencing transcription factor and pro-inflammatory cytokines, while simultaneously upregulating mu-opioid receptor (MOR) [104]. Emerging evidence highlights the pivotal role of circular RNAs in the pathogenesis of CIBP. A study profiling the expression landscape in the spinal cord of CIBP model rats identified multiple differentially expressed circRNAs, among which circSlc7a11 exhibited the most significant alteration. Notably, the overexpression of circSlc7a11 was found to inhibit tumor cell apoptosis and exacerbate the development of pain [105].

Currently, clinical research has predominantly focused on the direct eradication of tumor cells, leaving the management of CIBP—a primary and debilitating complication—relatively sidelined. Consequently, investigations into exosome-based interventions for cancer pain remain in their infancy. Nevertheless, CIBP represents the most prevalent and clinically challenging form of cancer pain, encompassing both primary bone tumors and the more frequent agonizing pain associated with skeletal metastases. In the pathophysiology of CIBP, the disruption of bone remodeling homeostasis is recognized as the core mechanism [106]. Tumor cells break the physiological coupling between osteoblasts and osteoclasts by secreting key factors such as the receptor activator of the nuclear factor κB ligand, leading to the pathological activation of osteoclasts. This process not only constructs an acidic microenvironment via proton (H^+^) secretion, which directly irritates sensory nerve endings within the bone marrow cavity, but also causes mechanical instability and breakthrough pain due to osteolytic destruction [107]. Concurrently, impaired osteoblast function further exacerbates bone remodeling disorders and tissue damage. This cytokine-mediated vicious “tumor–bone–nerve” cycle constitutes the biological foundation for the generation and maintenance of CIBP [108].

Notably, substantial research has accumulated regarding the role of exosomes in regulating bone remodeling. For instance, Mathieu et al. reported that exosomes derived from human prostate cancer cells (C4-2B and PC-3) carry the phospholipase D2 protein, which stimulates extracellular signal-regulated kinases 1 and 2 phosphorylation and significantly upregulates tissue-nonspecific alkaline phosphatase activity and osteogenic differentiation markers, thereby promoting osteoblast proliferation and differentiation [109]. Regarding osteolysis, Yuan et al. demonstrated that exosomes derived from breast cancer cells (SCP28) play a pivotal role in establishing the pre-metastatic niche, primarily through the horizontal transfer of miR-21 to osteoclasts, and clinical data further show elevated serum miR-21 levels in breast cancer patients, correlating with the severity of bone destruction [89]. Additional evidence supports the regulatory role of exosomes in bone remodeling within the context of bone cancer [110].

In summary, accumulating evidence suggests that exosomes serve as critical messengers mediating the imbalance between osteoblasts and osteoclasts within the bone microenvironment. Targeting these exosomal regulatory pathways has significant therapeutic potential, not only for inhibiting bone metastasis but also for alleviating CIBP by breaking the pathological vicious cycle.

### 5.4. Bidirectional Exosomal Crosstalk Between Neurons and Non-Neuronal Cells

The current literature on the pathological role of exosomes in pain has predominantly focused on the unidirectional modulation of neurons by non-neuronal cells, such as macrophages, microglia, and astrocytes. However, the exosome-mediated transmission of pain signals is defined by a sophisticated bidirectional dialogue rather than a simple unidirectional secretion. During the onset of pain, exosomes derived from non-neuronal cells activate neurons. Subsequently, these activated neurons actively release specific exosomes acting as “retrograde” signals to stimulate non-neuronal cells. This feedback forces an alteration in the composition of non-neuronal exosomes, thereby establishing a vicious cycle that perpetuates pain.

Initially, activated non-neuronal cells release exosomes encapsulated with algogenic cargos, including specific miRNAs and metabolic enzymes. These exosomes target pre- or post-synaptic neurons to suppress inhibitory Gama-aminobutyric acid receptors or potentiate excitatory N-methyl-D-aspartic acid receptor function, resulting in neuronal disinhibition and hyperexcitability. In response, activated neurons release “feedback” exosomes to prevent glial cells from returning to a resting state. For instance, Simeoli et al. demonstrated that injured DRG neurons release exosomes enriched with miR-21. Upon phagocytosis by macrophages, these exosomes activate TLR8, promoting a transition toward a pro-inflammatory phenotype, thereby highlighting the critical role of miR-21 in sensory neuron–macrophage communication following peripheral nerve injury [64]. Similarly, neuronal exosomes transfer miR-124a to astrocytes, selectively upregulating glutamate transporter-1 protein expression. By enhancing glutamate uptake and preventing excitotoxicity, this pathway modulates synaptic stability, offering a potential mechanism by which to reverse central sensitization in chronic pain states [111].

This reciprocal interaction creates a positive feedback loop: activated macrophages or glia escalate the secretion of pro-inflammatory exosomes, which further amplifies neuronal excitability. Higher neuronal excitability, in turn, generates stronger feedback signals to glia, inducing the release of more algogenic exosomes and ultimately maintaining central sensitization. In summary, while exosomes play an indispensable role in signal transduction between non-neuronal cells and nociceptors, the feedback regulation of non-neuronal cells by nociceptors warrants further investigation. Future studies are essential to fully characterize this bidirectional exosomal modulation in the pathogenesis of chronic pain.

### 5.5. Sex Differences as a Key Factor in Exosomal Pain Therapy

Historically, pain processing mechanisms were considered to be evolutionarily conserved across sexes. However, recent breakthrough studies have fundamentally overturned this assumption, revealing distinct cellular immune pathways governing the maintenance of neuropathic pain in males versus females. This fundamental sexual dimorphism within pain transmission pathways represents a critical biological variable that profoundly influences translational efficacy, yet it remains grossly underestimated in current preclinical research. Pioneering work by Sorge et al. demonstrated that neuropathic pain in male mice hinges on the activation of the TLR4-p38 MAPK signaling pathway in spinal microglia. Conversely, the development of mechanical allodynia in females bypasses microglial involvement, relying instead on T cells of the adaptive immune system and peroxisome proliferator-activated receptor gamma signaling [112]. Subsequent investigations have further corroborated that, compared to female mice in the SNI model, male spinal microglia exhibit more extensive differential gene expression changes, reflecting a more robust inflammatory activation profile [113]. This conclusion is strongly supported by transcriptomic evidence from Tansley et al. Employing RNA sequencing analysis on spinal microglia from models of nerve injury and CIPN, their study revealed that while both sexes upregulated genes associated with inflammation, phagosome, and lysosome activation, only male mice exhibited a prominent global transcriptional shift. This unique molecular signature indicates that male microglia undergo a specific state of acute activation, whereas females do not experience an equivalent degree of transcriptional reprogramming [114].

These disparities in cellular and molecular mechanisms directly challenge the universal applicability of existing exosome-based therapies. Given that the vast majority of current therapeutic strategies aim to deliver specific cargoes to suppress microglial polarization toward the M1 pro-inflammatory phenotype, this treatment paradigm targeting the “microglia–neuroinflammation” axis is likely to be highly effective in males but may show compromised efficacy in females due to target absence or signaling pathway mismatches. Consequently, future translational research on exosomes must abandon the traditional “male-as-norm” paradigm and mandate the inclusion of sex-balanced models. Only by elucidating the regulatory specificity of exosomes within sexually dimorphic immune microenvironments can we achieve the goal of precision analgesia.

## 6. Exosome Application for Pain

### 6.1. Exosomes as Delivery Carriers for Pain Management

MSCs exhibit immunomodulatory, anti-inflammatory, and nutritional properties [115]. In recent years, MSC transplantation has offered a potential therapeutic strategy for managing pain, especially neuropathic pain associated with nerve injury [116,117]. However, there are limitations associated with the use of MSCs for pain relief. First of all, MSC processing and storage are not convenient. Second, transplanted MSCs exhibit limited homing efficiency to target tissues and poor viability in inflammatory environments. Third, potential risks associated with MSC therapy include pulmonary embolism and tumorigenesis. Third, potential risks associated with MSC therapy include pulmonary thrombosis and endogenous tumor formation [118]. MSC-derived exosome transplantation offers several advantages over MSC therapy, including non-immunogenicity, non-tumorigenicity, and improved ease of storage and transportation [117]. Utilizing exosomes for pain management represents a promising novel therapeutic approach. This cell-free strategy recapitulates the therapeutic efficacy of MSCs while circumventing their inherent limitations [37]. Exosomes are acknowledged as a viable therapeutic approach or drug delivery system for the transfer of diverse substances, such as proteins and regulatory genes, to target cells. The non-immunogenic characteristics of these nanovesicles enable them to safeguard their cargoes from serum proteases and immune reactions [37,119,120,121].

OA affects multiple joint issues and is associated with severe pain, inflammation, and chronic cartilage degeneration [122]. Gene therapy can enable the precise expression of therapeutic proteins to enhance cartilage regeneration in osteoarthritis. The dense, avascular cartilage, rich in aggrecan and glycosaminoglycans (GAGs), presents a challenge for the effective transport of substances to chondrocytes due to its negative charge [123,124]. To address these issues, Zhang et al. developed surface-modified cartilage-targeting exosomes as non-viral carriers for gene therapy. Cationic exosomes traversed the full thickness of early-stage arthritic mouse cartilage due to weak-reversible ionic interactions with GAGs and effectively transported the encapsulated enhanced green fluorescent protein eGFP mRNA to chondrocytes located in the deep layers of tissue to facilitate cartilage regeneration [125].

**Figure 4:**
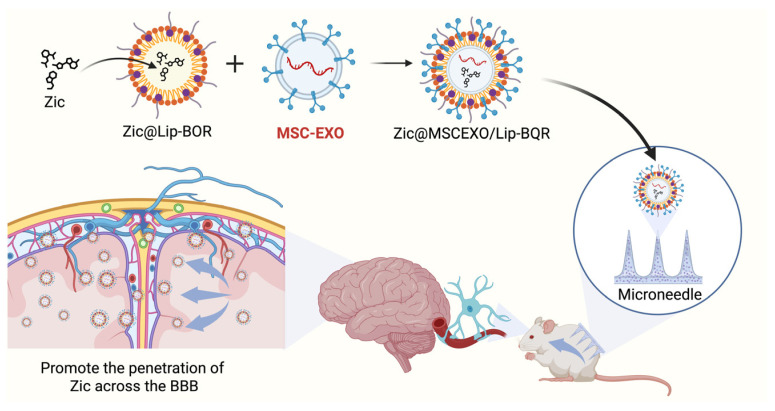
Exosomes are used for the delivery of analgesic drugs through the BBB. BOR-modified ZIC-loaded LIPs were fused with the exosomal membrane and converted into gelatin-based soluble MNs, forming a ZIC drug delivery system, which has proven effective in promoting ZIC penetration through the BBB. (Created in Biorender. Wen, Y. (2026) https://BioRender.com/p01mh0g).

Due to the limitations of the blood–brain barrier (BBB), delivering drugs to the brain has been a major challenge in the treatment of central nervous pain. As a new type of natural drug delivery carrier, exosomes can effectively cross the main biological barriers including cytoplasmic membrane and blood–brain barrier [126]. Ziconotide (ZIC) functions as an N-type calcium channel antagonist, utilized for the management of severe chronic pain that is either intolerable or inadequately responsive to alternative treatments, including intrathecal morphine and systemic analgesics. Intrathecal injection is the sole administration route for ZIC as it functions exclusively within the brain and cerebrospinal fluid [127,128,129]. Nevertheless, the injection equipment and devices utilized for intrathecal administration may pose specific risks. Song et al. combined prepared borneol (BOR)-modified liposomes (LIPs) with exosomes derived from MSCs and loaded ZIC to create microneedles (MNs). Then, the authors demonstrated that exosome-mediated delivery significantly enhanced the translocation of ZIC across the BBB and into the cerebrospinal fluid (Figure 4). To assess the local analgesic effects of MNs, behavioral pain sensitivity to thermal and mechanical stimulation was evaluated in animal models. The results indicated that the MN#ZIC@MSCEXO/LIP-BOR administration system effectively delivers ZIC to the cerebrospinal fluid and demonstrated analgesic efficacy across various pain models, including peripheral nerve injury, diabetes-induced neuropathic pain, chemotherapy-induced pain, and ultraviolet-B radiation-induced neurogenic inflammatory pain. This study on the delivery of drugs provides a safe and effective administration for chronic pain treatment, as well as demonstrates great potential for clinical application of exosomes [130].

The versatility of exosomes to be engineered for carrying different molecules has been demonstrated in multiple studies and under varied experimental conditions [32]. In sensory phenotypes where irritable nociceptors cause chronic pain, exosomes cargoes might be controlled to transport tiny peptides or anti-inflammatory compounds to modify the function of thermoreceptors, mechanoreceptors, and chemoreceptors. For instance, curcumin encapsulated with exosomes, in comparison to free curcumin, exhibited strong anti-inflammatory activity in vitro and in vivo against lipopolysaccharide [131]. In addition, exosomes, as natural drug carriers, can be engineered to achieve targeted drug delivery, elevated drug concentration at the disease site, and enhanced therapeutic efficacy, minimizing the adverse effects associated with systemic drug administration [132]. Certain researchers have utilized exosomes derived from MSCs as repair vectors to create “G-C” DNA tetrahedral nanostructures on their surfaces, aiming to mitigate doxorubicin-induced toxicity [133]. Additionally, exosomes can be engineered with myocardial-targeting peptides to mitigate chemotherapy-induced cardiotoxicity [134]. These studies suggest the use of modified exosomes to deliver analgesics to reduce dosage and side effects.

### 6.2. Advantages of Exosomes over Existing Analgesic Therapies and Challenges in Translation

Compared with current pharmacological interventions, exosomes offer unique therapeutic dimensions. Traditional medications, such as non-steroidal anti-inflammatory drugs and opioids, primarily achieve transient symptomatic relief by blocking pain signaling pathways. However, these therapies lack the capacity for substantial tissue repair and are often associated with severe adverse effects, including addiction and gastrointestinal toxicity. In contrast, as bioactive vehicles, exosomes can deliver specific growth factors and miRNA cargoes to promote cartilage regeneration, neural repair, and the remodeling of the immune microenvironment. By exerting these potential “disease-modifying” effects, exosomes can fundamentally intervene in pathological processes to achieve long-lasting analgesia [135]. Furthermore, the BBB remains a significant bottleneck in the treatment of central pain. However, exosomes exhibit superior biobarrier-penetrating capabilities due to their nanoscale size and natural lipid bilayer structure. This allows them to serve as highly efficient delivery systems, precisely transporting therapeutic molecules to spinal or cerebral targets, thereby offering a promising intervention strategy for refractory central pain [136].

As a “cell-free” therapeutic strategy, exosomes significantly circumvent the risks associated with live cell transplantation (such as MSCs), including potential tumorigenicity, vascular embolism, and immune rejection. This exceptional biosafety profile, combined with their physicochemical stability (e.g., resistance to freeze–thaw cycles and ease of storage), makes exosomes better suited for development into standardized “off-the-shelf” pharmaceutical products [137,138].

Despite these dual advantages in efficacy and safety, exosomes still face feasibility challenges in clinical translation. Compared to small-molecule chemical drugs, conventional medications currently maintain a significant edge in terms of large-scale production and cost-effectiveness. The large-scale manufacturing of good manufacturing practice (GMP)-grade exosomes involves complex upstream cell culture and downstream purification processes. The high production costs and the lack of unified quality control standards constitute substantial barriers to clinical entry [139]. Consequently, establishing standardized, high-yield manufacturing processes remains a critical issue that must be addressed to facilitate the clinical application of exosomes.

## 7. Clinical Trials and Challenges of Exosomes in Pain Management

### 7.1. Clinical Investigations of Exosomes for Pain Treatment

Currently, the clinical translation of exosomes in pain management is in its nascent stage, primarily focusing on the treatment of NP and low back pain. Preliminary evidence indicates a favorable safety profile and several potential clinical benefits. Prospective studies conducted by Phillips et al. and Wilson et al. demonstrated that the administration of BM-MSC-derived exosomes via epidural or facet joint injection significantly improved pain scores and functional disability during short-term follow-up (1–3 months). These findings not only corroborate that exosomes can recapitulate the therapeutic benefits of MSC therapy but also effectively circumvent the potential risks of immune rejection and tumorigenicity associated with live cell transplantation [140]. Additionally, although a registered clinical trial investigating the intradiscal injection of peripheral blood-derived exosomes has been completed, specific data have not yet been publicly released. In the realm of translational research for diabetic neuropathic pain (DNP), the potential of exosomes as vehicles for molecular cargo transport has garnered significant attention. Clinical analysis revealed significantly elevated levels of lncRNA NONRATT021972 in the plasma of DNP patients, which were positively correlated with TNF-α-mediated inflammatory responses; subsequent animal experiments confirmed that silencing this lncRNA significantly alleviated pain behaviors and neuroinflammation. This finding suggests that the targeted regulation of specific circulating lncRNAs carried by exosomes may represent a novel strategy for future DNP interventions [141].

Despite encouraging preclinical data, the translation of exosome therapy to clinical application remains beset by multiple hurdles, characterized primarily by limitations in trial design and the incompleteness of data reporting. We have summarized the clinical trials evaluating exosomes as analgesic agents in Table 4. First, existing clinical evidence stems largely from single-center, open-label, early-stage exploratory studies; thus, we lack high-quality evidence-based medical support. For instance, the clinical trial conducted by Akhlaghpasand et al. (IRCT20200502047277N1) primarily aimed to assess safety and did not include a placebo control group. Given that pain is a subjective experience highly susceptible to psychological factors, the absence of blinding and control groups makes it difficult to disentangle confounding factors such as the “placebo effect” and the natural history of the disease, thereby preventing the verification of the true efficacy of exosomes. Furthermore, the sample sizes in this phase of trials are severely insufficient to provide adequate statistical power to evaluate the precise clinical benefits of intrathecal exosome injection [142]. Secondly, the “opacity” of clinical data further impedes scientific assessment. For example, clinical trial NCT04849429, designed to evaluate the safety and efficacy of intradiscal injection of autologous peripheral blood exosomes for low back pain, is listed as “completed”; however, no results have been disclosed on ClinicalTrials.gov or in peer-reviewed journals to date. Similar “data black holes” exist in other trials that are recruiting (NCT05152368) or have been terminated (NCT04202783). This publication bias makes it difficult for the academic community to comprehensively assess the risks and benefits of this therapy.

In summary, current clinical research on exosome therapy for pain is characterized by being “sporadic, early-stage, and non-standardized.” Despite demonstrating superior analgesic potential in preclinical models, a substantial gap remains before exosomes can become a mature, routine analgesic agent, due to the lack of support from large-scale, multi-center, randomized double-blind controlled trials, the absence of unified GMP standards for preparation, and the opacity of clinical data reporting. Future translational research urgently requires the establishment of strict quality control standards and the implementation of rigorously designed phase II/III clinical trials to confirm long-term efficacy and safety within the framework of evidence-based medicine.

### 7.2. Unique Properties of Exosomes as Ideal Pain Biomarkers

The hallmark characteristics of clinical pain disorders, namely, persistence and subjectivity, pose significant challenges to objective assessment. Traditional scoring systems often fail to effectively differentiate between the emotional and somatosensory dimensions of pain, frequently leading to biased assessments. While neuroimaging technologies can provide real-time radiographic evidence [143], fluid-based biomarkers offer deeper insights into the underlying pathological mechanisms, states of neural activity, and individual susceptibility associated with pain [143]. In this context, exosomes have emerged as a novel liquid biopsy tool with immense application potential.

Current clinical evidence suggests that exosomes hold promise not only as predictive biomarkers for persistent pain but also as tools for monitoring therapeutic responses. Regarding prognostic assessment, Moen et al. demonstrated that the dynamic alteration of exosomal miR-223-3p is closely associated with the risk of developing chronic pain following lumbar disk herniation [144]. Specifically, in the recovery group (defined as a visual analog scale score reduction > 2 over one year), miR-223-3p levels exhibited a “rise-then-fall” trend, whereas no significant changes were observed in the persistent pain group. This suggests that miR-223-3p may serve as a molecular signature for predicting patient outcomes. In terms of disease diagnosis, significantly upregulated levels of circulating miR-140-3p were identified in chronic myeloid leukemia patients suffering from musculoskeletal pain [145]. Similarly, CRPS patients exhibited distinct differential exosomal expression profiles [14]. Furthermore, regarding therapeutic monitoring, exosomal miRNA expression levels demonstrate responsive changes to treatment. For instance, specific miRNAs that were initially upregulated in the blood of osteoarthritis patients were significantly downregulated following mud-bath therapy, a change that coincided with improvements in visual analog scale scores [146]. Another independent study confirmed that the analgesic response to ketamine treatment in CRPS patients is correlated with specific miRNA expression profiles [147]. Collectively, this evidence indicates that by identifying specific molecular cargo profiles highly correlated with pain states, exosomes can effectively distinguish between pathological and healthy conditions, thereby facilitating the early diagnosis and therapeutic monitoring of pain.

Compared with traditional serum biomarkers, exosomes exhibit significant technical advantages in pain diagnosis, a potential that has already been validated in commercial diagnostic assays for diseases such as prostate cancer [148]. First, traditional serum biomarkers (particularly free RNA) are highly susceptible to degradation by RNases or proteases present in body fluids, thereby compromising detection sensitivity. In contrast, the robust lipid bilayer structure of exosomes provides a natural barrier that effectively protects the encapsulated nucleic acids and proteins. This protection ensures high stability during freeze–thaw cycles or long-term storage, guaranteeing the accuracy of quantitative analyses [148]. Second, blood represents a heterogeneous pool of secretions from multiple tissues, where traditional biomarkers are often difficult to trace back to their origin due to the “dilution effect.” Uniquely, the exosomal surface retains specific antigens from parental cells. Leveraging immuno-affinity capture technologies allows for the specific enrichment of nervous system-derived exosomes from complex peripheral blood. This is particularly crucial for chronic pain states dominated by central mechanisms as it effectively excludes interference from peripheral tissues and significantly improves the diagnostic signal-to-noise ratio [149]. Finally, while pain is often accompanied by central sensitization, the BBB typically restricts the release of central pathological information into the periphery. However, owing to their nanoscale size and lipid properties, exosomes can cross the BBB bidirectionally, transporting algogenic molecules from spinal dorsal horn glial cells or DRG neurons into the peripheral circulation [126]. Consequently, the detection of peripheral blood exosomes serves as a proxy for the non-invasive monitoring of the central microenvironment.

Despite these promising prospects, the clinical translation of exosomes as pain biomarkers remains hindered by substantial objective hurdles. The field currently lacks a unified “gold standard” for isolation techniques. The significant variability in exosomal subpopulations and purity obtained via different methods (e.g., ultracentrifugation vs. commercial precipitation kits) impedes the reproducibility and cross-comparison of data between laboratories [149,150]. In addition, circulating exosomes exhibit a high degree of heterogeneity; those genuinely associated with pain pathology may constitute only a minute fraction of the total population. In the absence of immuno-enrichment using specific antibodies, these faint disease-specific signals are prone to being masked by the vast background noise, thereby compromising diagnostic specificity and sensitivity.

### 7.3. Current Limitations of Exosomes as Diagnostic Tools for Pain

Beyond clinical diagnosis, another core challenge in pain management lies in prognostic assessment: specifically, how to accurately predict which acute pain patients will transition to chronic pain, thereby enabling early risk stratification. A current limitation is the scarcity of reliable longitudinal evidence validating the causal association between exosomal biomarkers and pain chronification. Consequently, bridging this critical knowledge gap is urgent. Future research should prioritize prospective, longitudinal cohort designs, implementing rigorous time-window sampling strategies (e.g., targeting the acute phase of 24–72 h post-injury and early follow-up points), and establishing clear chronic pain endpoints (e.g., at 3, 6, and 12 months), supplemented by multi-center external validation to ensure generalizability.

As a “liquid biopsy” tool, exosomes offer a unique advantage in capturing the dynamic trajectory of neuro-immune interactions in real time. By monitoring the transition of exosomal molecular profiles from an initial pro-reparative state to a persistent, pathological pro-inflammatory and synaptic remodeling phenotype, we may be able to identify critical therapeutic windows prior to the consolidation of pain memory. This would allow for early intervention to interrupt the pathological progression from acute to chronic pain.

Another unmet and pressing need in clinical pain medicine is etiology-oriented differential diagnosis, particularly when clinical manifestations are ambiguous or overlapping among neuropathic, inflammatory, and cancer pain. Existing studies are largely confined to a binary comparison mode of “single disease group vs. healthy controls,” lacking lateral comparisons across different pain subtypes. However, since inflammatory responses and tissue injury signaling pathways are widely shared across multiple pain etiologies, mere comparisons with healthy individuals often fail to identify highly specific biomarkers.

Therefore, to overcome this pathophysiological overlap, future diagnostic studies must incorporate etiology-matched disease controls—that is, they must directly compare exosomal profiles among patients with inflammatory, neuropathic, and cancer pain within the same research framework. This design strategy will facilitate the isolation of shared background signals and the identification of specific “molecular fingerprints” capable of precisely distinguishing specific pain subtypes.

## 8. Conclusions and Perspectives

Pain is a complication associated with various diseases, which seriously affects the quality of life of patients. At present, the effect of the drugs used in clinical treatment of pain is unsatisfactory, and the main problems are the short duration of analgesia and various adverse reactions [151]. Exosomes serve as communication carriers between cells and offer distinct advantages in pain treatment, potentially enhancing therapeutic outcomes.

Exosomes’ intrinsic “homing” ability suggests their potential utility in drug delivery. Exosomes from various cellular origins can migrate directionally through specific membrane surface receptors or extracellular matrix binding proteins, allowing for rapid recognition, uptake, and functionality by targeted recipient cells [152]. Liu et al. investigated the interaction between hepatocellular carcinoma (HCC) and adipocytes to develop a hybrid adipocyte-derived exosome platform containing the anti-cancer prodrug docetaxel, which serves as a targeted vehicle for HCC with significant antitumor efficacy. In vitro and in vivo experiments demonstrate that hybrid exosomes strengthen the bioactivity of the prodrug, extend its circulation in the bloodstream, and effectively inhibit tumor growth by selectively targeting hepatocellular carcinoma tumor cells with minimal side effects [153]. Similarly, analgesic drugs can also be delivered by exosomes, which prolongs drug retention in the body and facilitates sustained analgesic effects. Additionally, the targeting capability of exosomes minimizes potential adverse reactions.

Intrathecal drug injection is currently an internationally recognized effective means of treating intractable pain, but its operation is complicated, most patients need multiple doses, and repeated puncture can easily lead to reinfection [154]. Exosomes can cross the BBB due to their cellular architecture, allowing for the delivery of analgesic drugs into the cerebrospinal fluid for central analgesia, hence addressing issues related to intrathecal injection [155].

Furthermore, numerous studies have demonstrated the role of exosomes in various pain-related processes; however, the molecular mechanisms underlying their action remain incompletely understood at present. Consequently, additional research with a substantial sample size is essential to address these deficiencies, acquire comprehensive understanding of the significance of exosomes, and establish a reliable, standardized, and cost-effective laboratory technique. This procedure is critical for the clinical translation of exosomes, paving the way for precision, patient-centered medicine, with a revolutionary influence in terms of efficacy, safety, and cost-effectiveness.

## Figures and Tables

**Table 3:** Summary of exosomes and cancer pain.

Donor	Cargo	Animal	Disease Model	Recipient	Targets/Signaling Pathway	Ref.
NCTC 2472	-	-	-	DRG	ATX	[99]
HSC-3/OSC-20	-	Mouse	-	-	-	[100]
A549/NCI-H1299	let-7d-5p	Mouse	CIBP	DRG	OPRM1	[101]
DRG	miR-34c-5p	Mouse	CIBP	-	Cav2.3	[102]
-	miR-135-5p	Mouse	CIBP	Astrocyte	JAK2/STAT3	[103]
BMSCs	miR-5-9p	Mouse	CIBP	-	MOR	[104]
-	circSlc7a11	Rat	CIBP	-	-	[105]

**Table 4:** Clinical trials of exosome-based therapies for pain.

Trial ID	Condition	Participants	Exosomes Source	Route	Phase	Status
IRCT20200502047277N1	Neuropathic Pain	9	HUC-MSCs	Intrathecal injection	I	Completed
NCT05152368	Peripheral Neuropathic Pain	20	UC-MSCs	(Single dose: 300 μg)	I	Recruiting
NCT04849429	Neuropathic Pain	30	Evs derived from the peripheral blood	Intranasal instillation	I	Completed
NCT04202783	Trigeminal neuralgia	100	UC-MSCs	(Single dose: 8 × 10^10^ partcles)	-	Suspended

## Data Availability

No new data were created or analyzed in this study.

## Abbreviations

The following abbreviations are used in this manuscript:

ACS	Autologous conditioned serum
BBB	Blood–brain barrier
BMSCs	Bone marrow mesenchymal stem cells
BOR	Borneol
Cav2.3	Voltage-gated calcium channel 2.3
CCI-ION	Chronic constriction injury of the infraorbital nerve
CFA	Complete Freund’s adjuvant
CIBP	Cancer-induced bone pain
CIPN	Chemotherapy-induced neuropathic pain
COL10A1	Collagen type X alpha 1
CRPS	Complex regional pain syndrome
DMM	Destabilization of the medial meniscus
DNP	Diabetic neuropathic pain
DRGs	Dorsal root ganglions
ESCRT	Endosomal sorting complex required for transport
ESEs	Early sorting endosomes
Evs	Extracellular vesicles
GAGs	Glycosaminoglycans
GDNF	Glial cell line-derived neurotrophic factor
GMP	Good manufacturing practice
HCC	Hepatocellular carcinoma
hCS	Human conditioned serum
HECTD1	HECT domain E3 ubiquitin protein ligase 1
HUC-MSCs	Human umbilical cord mesenchymal stem cells
IBD	Inflammatory bowel disease
IL1R1	Interleukin 1 receptor type 1
IL-1β	Interleukin-1β
ILVs	Intraluminal vesicles
JAK2	Janus kinase-2
LIPs	Liposomes
LPA	Lysophosphatidic acid
LRP1	Lipoprotein receptor-related protein 1
LSEs	Late sorting endosomes
MNs	Microneedles
MOR	Mu-opioid receptor
MSC	Mesenchymal stem cell
MVBs	Multivesicular bodies
MVs	Microvesicles
MyD88	Myeloid differentiation primary response protein 88
NF-κB	Nuclear factor kappa-B
NLRP3	NOD-like receptor thermal protein domain-associated protein 3
NP	Neuropathic pain
OA	Osteoarthritis
OPRM1	Opioid receptor mu 1
p38 MAPK	p38 mitogen-activated protein kinase
RA	Rheumatoid arthritis
Rsad2	Radical S-adenosyl methionine domain-containing 2
S1P	Sphingosine-1-phosphate
SHED	Stem cells of human exfoliated deciduous teeth
SNI	Spared nerve injury
SNL	Spinal nerve ligation
SP1	Specificity protein 1
STAT3	Signal transducer and activator of transcription 3
STN	Spinal trigeminal nucleus
TLR2	Toll-like receptor 2
TLR3	Toll-like receptor 3
TLR4	Toll-like receptor 4
TLR8	Toll-like receptor 8
TRAF6	TNF receptor-associated factor 6
VEGFB	Vascular endothelial growth factor B
ZIC	Ziconotide
